# Target enrichment from a DNA mixture by oligoribonucleotide interference-PCR (ORNi-PCR)

**DOI:** 10.1093/biomethods/bpz009

**Published:** 2019-08-01

**Authors:** Toshitsugu Fujita, Daisuke Motooka, Hodaka Fujii

**Affiliations:** 1 Department of Biochemistry and Genome Biology, Hirosaki University Graduate School of Medicine, 5 Zaifu-cho, Hirosaki, Aomori, Japan; 2 Department of Infection Metagenomics, Genome Information Research Center, Research Institute for Microbial Diseases, Osaka University, 3-1 Yamadaoka, Suita, Osaka, Japan

**Keywords:** PCR, ORNi-PCR, oligoribonucleotide, genome editing, microbiome profiling, *16S rRNA* genes

## Abstract

Oligoribonucleotide (ORN) interference-PCR (ORNi-PCR) is a method that suppresses PCR amplification of target DNA in an ORN-specific manner. In this study, we examined whether ORNi-PCR can be used to enrich desirable DNA sequences from a DNA mixture by suppressing undesirable DNA amplification. ORNi-PCR enriched edited DNA sequences from a mixture of genomic DNA subjected to genome editing. ORNi-PCR enabled more efficient analysis of the types of insertion/deletion mutations introduced by genome editing. In addition, ORNi-PCR reduced the detection of *16S ribosomal RNA* (*16S rRNA*) genes in *16S rRNA* gene-based microbiome profiling, which might permit a more detailed assessment of populations of other *16S rRNA* genes. Enrichment of desirable DNA sequences by ORNi-PCR may be useful in molecular biology, medical diagnosis, and other fields.

## Introduction

Polymerase chain reaction (PCR) can specifically amplify target deoxyribonucleic acid (DNA) sequences and is widely used in various applications. However, primer sets designed to amplify a specific target DNA sequence by PCR may also amplify nontarget sequences. In such cases, blocking PCR with 3′-modified DNAs and artificial nucleic acids such as locked nucleic acids and peptide nucleic acids (PNAs) can be used to suppress undesirable amplification [1]. In this context, we previously showed that oligoribonucleotides (ORNs) of 17–29 bases in length can be used to block PCR and named the method ORN interference-PCR (ORNi-PCR) [2].

**Figure 1:**
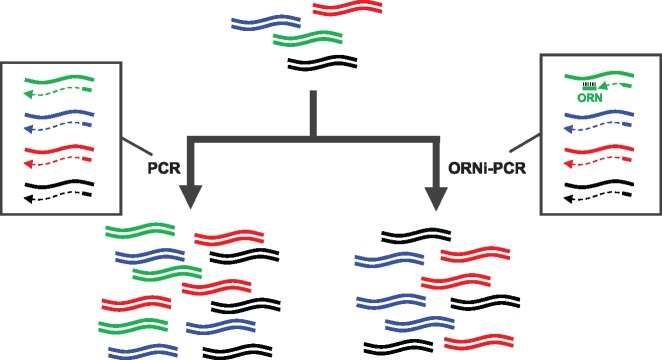
Schematic diagram showing enrichment of target DNA and suppression of the amplification of nontarget DNA by ORNi-PCR. An ORN designed to hybridize with a target site in a sequence (green) suppresses amplification across the sequence by blocking elongation by DNA polymerases in ORNi-PCR. Consequently, target DNA sequences (blue, red, and black) can be enriched.

ORNi-PCR suppresses the amplification of a DNA sequence of interest using an ORN complementary to a target site to inhibit DNA amplification by DNA polymerases (Fig 1). ORNs can be flexibly designed and economically synthesized, offering advantages over artificial nucleic acids. In addition, ORNs can function as PCR blockers without the need for any chemical modifications. However, because DNA polymerases with 5′-3′ exonuclease activity (i.e. pol I types such as *Taq*) may degrade ORNs during DNA extension, those without 5′-3′ exonuclease activity (i.e. α-types such as KOD or *Pfu*) should be used for ORNi-PCR. ORNi-PCR can be applied to the detection of nucleotide differences. Indeed, we previously demonstrated that ORNi-PCR can discriminate nucleotide differences introduced by genome editing or single-nucleotide mutations in cancer cells [3, 4]. However, it remains unclear whether ORNi-PCR can enrich desirable DNA sequences from a DNA mixture by suppressing undesirable DNA amplification (Fig. 1).

In this study, we attempted to enrich desirable DNA sequences from a mixture of DNA by ORNi-PCR. First, suppressing amplification of a wild-type (WT) DNA sequence, we succeeded in enriching DNA sequences containing insertion/deletion (indel) mutations introduced by genome editing from a genomic DNA (gDNA) mixture. Next, we succeeded in lowering detection of a target *16S ribosomal RNA* (*16S rRNA*) gene from a bacterial genus in *16S rRNA* gene-based microbiome profiling. Thus, ORNi-PCR could be utilized for more effective and lower-cost analysis of indel mutations introduced by genome editing, and higher resolution analysis of *16S rRNA* gene-based microbiome profiling.

## Materials and methods

### Oligonucleotides

ORNs purified by high-performance liquid chromatography were purchased from FASMAC (Kanagawa, Japan). ORNs and primers are listed in Supplementary Table S1.

### Genome editing

HCT116 cells were cultured in McCoy’s 5A medium (Thermo Fisher Scientific, Waltham, MA, USA) supplemented with 10% fetal bovine serum. For genome editing of the human *thymocyte nuclear protein 1* (*THYN1*) locus, HCT116 cells (4 × 10^5^) were transfected with a Cas9 expression plasmid (2 µg, Addgene #41815; a kind gift from Dr George M. Church) [5] and a single guide RNA (sgRNA) expression plasmid targeting the human *THYN1* locus (2 µg) [3] using Lipofectamine 3000 (Thermo Fisher Scientific). After 3 days, cells were collected for extraction of gDNA. For genome editing of the human *CDKN2A (p16)* locus, HCT116 cells (4 × 10^5^) were transfected with the Cas9 expression plasmid (2 µg) and a sgRNA expression plasmid targeting the human *CDKN2A (p16)* locus (2 µg, sgRNA_lef5) [6] using Lipofectamine 3000 (Thermo Fisher Scientific). After 3 days, cells were collected for extraction of gDNA.

### DNA preparation

gDNA was extracted from HCT116 cells and *Escherichia coli* (*E. coli*) DH5α (Competent Quick DH5α; Toyobo, Osaka, Japan) using a Quick-DNA Universal Kit (Zymo Research, Irvine, CA, USA). A 16S rRNA_Mega plasmid containing the V1−V2 region of *Megamonas* sp. *16S rRNA* was designed and purchased from Eurofins Genomics (Tokyo, Japan). Human stool DNA was extracted using a PowerSoil DNA Isolation Kit (MO BIO Laboratories, Carlsbad, CA, USA).

### ORNi-PCR

Two-step ORNi-PCR was performed using KOD-Plus-Ver. 2 (Toyobo) as described previously [4] and in Supplementary Protocol. Briefly, reactions were carried out with an initial denaturation at 94°C for 2 min, followed by 30–34 cycles at 98°C for 10 s and 50°C–74°C for 60–90 s. ORNi-PCR products were electrophoresed on 1−2% agarose gels and, if necessary, subjected to DNA cloning and/or DNA sequencing analysis. DNA sequencing data were analyzed using Applied Biosystems Sequence Scanner Software v2.0 (Thermo Fisher Scientific). Alternatively, purified ORNi-PCR products were subjected to next-generation sequencing (NGS) analysis.

#### *16S rRNA* gene-based microbiome profiling

NGS analysis was performed as described previously [7]. Briefly, each library was prepared using two-step PCR with a primer set targeting the V1−V2 region of *16S rRNA* genes. Paired-end sequencing was performed using MiSeq (Illumina, San Diego, CA, USA). The relative abundance of bacteria in each sample was calculated by QIIME v1.9.1 (http://qiime.org/).

## Results and discussion

### Enrichment of genome-edited DNA sequences by ORNi-PCR

**Figure 2:**
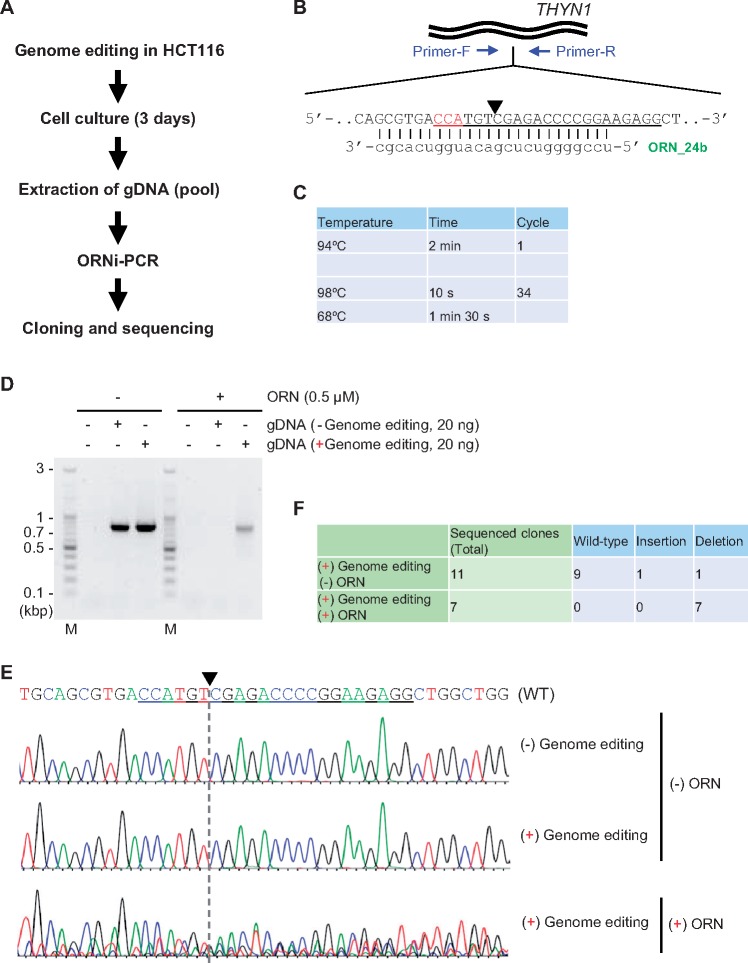
Enrichment of genome-edited DNA sequences by ORNi-PCR. (**A**) Schematic diagram of the analysis of genome-edited DNA sequences by ORNi-PCR. (**B**) Target position for genome editing and the sequence of ORN_24b targeting the human *THYN1* locus. The forward sequence of gDNA is shown. The CRISPR recognition sequence and cleavage site are underlined and indicated by an arrowhead, respectively. The protospacer adjacent motif is shown in red. (**C**) Conditions for two-step ORNi-PCR. (**D**) Results of ORNi-PCR. M, molecular weight markers. (**E**) DNA sequencing analysis of PCR and ORNi-PCR products. PCR and ORNi-PCR products in (D) were purified and subjected to Sanger sequencing using a forward primer. (**F**) Summary of different types of indel mutations. PCR and ORNi-PCR products in (D) were cloned into plasmids, and their DNA sequences were analyzed by Sanger sequencing.

Genome editing is an important biotechnological approach in various fields including molecular biology and medical diagnosis. To analyze the types of indel mutations introduced by genome editing, DNA sequences across a target site are amplified by PCR and traditionally analyzed by Sanger sequencing after DNA cloning. In this context, suppressing the amplification of nonedited (WT) DNA sequences can reduce cost and effort for such analyses, especially if genome editing is not effective. We, therefore, examined whether ORNi-PCR could suppress amplification of a WT DNA sequence from a pool of gDNA extracted from cells subjected to genome editing. To this end, we performed genome editing on the human *THYN1* locus in HCT116 cells using the clustered regularly interspaced short palindromic repeats (CRISPR) system [8, 9]. In this regard, we previously used ORNi-PCR to screen genome-edited cells [3] and succeeded in performing CRISPR-mediated genome editing at the human *THYN1* locus in Raji cells. In this study, we targeted the human *THYN1* locus as a model locus and used the same CRISPR components for genome editing. After genome editing, gDNA was extracted from the genome-edited cell pool and subjected to ORNi-PCR (Fig. 2A). We amplified DNA sequences across the CRISPR target site by PCR in the presence or absence of ORN_24b [3], a 24 base ORN complementary to a sequence around the cleavage site of the CRISPR system (Fig. 2B and C). In this regard, we previously demonstrated that two-step ORNi-PCR with ORN_24b can discriminate even a single-nucleotide difference from the WT sequence at an annealing and elongation temperature of 68°C [3]. As shown in Fig. 2D, when gDNA from WT HCT116 cells was used as template, amplification of the *THYN1* locus was completely suppressed in the presence of ORN_24b. By contrast, following genome editing, the *THYN1* locus was amplified even in the presence of ORN_24b. These results imply successful genome editing of the CRISPR target site. To confirm genome editing events, we directly sequenced the amplicons presented in Fig. 2D, and only WT *THYN1* sequencing signals were detected from PCR amplicons when ORN_24b was not added in the PCR mix, while sequencing signals different from the WT sequence were detected from the ORNi-PCR amplicon (Fig. 2E). In addition, the WT *THYN1* sequencing signal was not observed among sequence signals from the ORNi-PCR amplicon (Fig. 2E), suggesting that most of the detected sequencing signals were derived from edited DNA sequences.

We next cloned these PCR and ORNi-PCR amplicons and sequenced each clone by Sanger sequencing. In all seven ORNi-PCR clones, *THYN1* sequences were edited, whereas only 2 of 11 PCR clones were edited (Fig. 2F and Supplementary Fig. S1). These results suggest that ORNi-PCR can be used to confirm the presence of edited DNA sequences (i.e. successful genome editing) and enrich edited DNA sequences. In this context, NGS analysis can be employed instead of DNA cloning followed by Sanger sequencing for more comprehensive sequencing analysis. ORNi-PCR may reduce cost and effort for analysis of types of indel mutations introduced by genome editing.

We also applied this ORNi-PCR system to another locus. We performed genome editing of the human *CDKN2A (p16)* locus in HCT116 cells using the CRISPR system and evaluated the results (Supplementary Fig. S2A and B). In this experiment, we used crRNA_lef5 [6], a CRISPR RNA (crRNA) complementary to the target site of the CRISPR system, for ORNi-PCR. In this regard, we previously showed that crRNAs can be used for ORNi-PCR instead of ORNs [3]. When the target *CDKN2A (p16)* locus was successfully edited, the target sequence was amplified even in the presence of crRNA_lef5 (Supplementary Fig. S2C and D). We sequenced the amplicons presented in Supplementary Fig. S2D, and various sequencing signals were detected from the ORNi-PCR amplicon (Supplementary Fig. S2E). Thus, crRNA can also be utilized for enrichment of edited DNA sequences by ORNi-PCR. Notably, the WT sequence signal could be traced among DNA sequencing signals from the ORNi-PCR amplicon (Supplementary Fig. S2E), suggesting that suppression of the WT sequence may be incomplete, and further optimization of the experimental conditions may be required for crRNAs.

### Suppression of target *16S rRNA* amplification by ORNi-PCR

**Figure 3:**
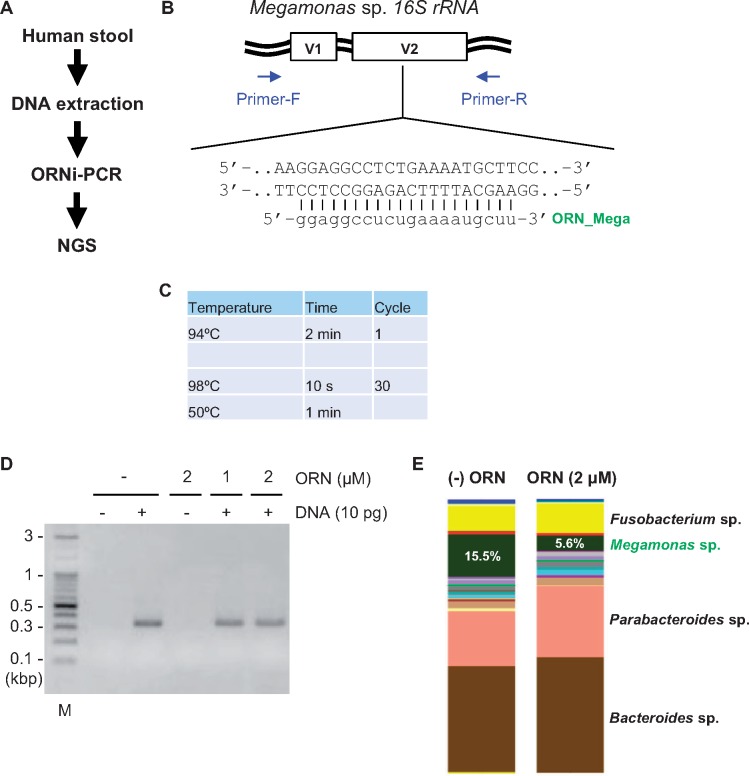
Suppression of target *16S rRNA* amplification by ORNi-PCR. (**A**) Schematic diagram of the analysis of *16S rRNA* by ORNi-PCR. (**B**) Target position of *16S rRNA* and the sequence of the ORN_Mega targeting the *Megamonas* sp. *rRNA* gene. Forward and reverse sequences of gDNA are shown. The V1 and V2 regions of *16S rRNA* were amplified using a common bacteria primer set. (**C**) Conditions for two-step ORNi-PCR. (**D**) Results of ORNi-PCR with human stool DNA. M, molecular weight markers. (**E**) Results of NGS analysis. PCR and ORNi-PCR products in (D) were purified and subjected to NGS analysis.

For microbiome profiling, variable regions of bacterial *16S rRNA* genes are amplified and analyzed by NGS. The human gut microbiome has been previously analyzed to examine the effects of changes in the gut microbiome on human health. Several bacterial genera are dominant in the human gut bacterial population and are abundantly detected by *16S rRNA* gene-based microbiome profiling [10]. If amplification of *16S rRNA* genes from such dominant genera can be suppressed by ORNi-PCR, *16S rRNA* genes from other less dominant genera might be more abundantly amplified and analyzed in more detail without increasing NGS read numbers (Supplementary Fig. S3). In this study, we therefore examined whether ORNi-PCR could suppress amplification of a *16S rRNA* gene from a target bacterial genus in *16S rRNA* gene-based microbiome profiling of a human gut microbiome (Fig. 3A). To this end, the V1−V2 regions of bacterial *16S rRNA* genes were amplified from DNA extracted from a human stool sample with bacteria-specific primers 27Fmod and 338R and sequenced by NGS (Supplementary Fig. S4A and B). As shown in Supplementary Fig. S4C, *16S rRNA* derived from *Megamonas* sp. was detected as one major bacterial population, accounting for 16.0% of the total population, mostly from a single *Megamonas* sp. V1−V2 sequence (Supplementary Table S2). *Bacteroides* sp. *16S rRNA* genes were more abundantly detected but were derived from some major species (i.e. V1−V2 sequences; Supplementary Table S2).

As a feasibility study, we attempted to suppress amplification of the *Megamonas* sp. *16S rRNA* sequence by ORNi-PCR by designing and testing a 20 base ORN_Mega targeting *the Megamonas* sp. *16S rRNA* gene (Fig. 3B). We first examined whether the designed ORN could suppress DNA amplification from a cloned *Megamonas* sp. *16S rRNA* gene (plasmid 16S rRNA_Mega; Supplementary Fig. S5A and B). ORN_Mega suppressed DNA amplification from the cloned DNA at annealing and elongation temperatures <55°C, and 50°C was most effective with 2 μM ORN (Supplementary Fig. S5C and D). To examine the specificity of ORN_Mega, we performed ORNi-PCR with gDNA extracted from *E. coli* DH5α. The *16S rRNA* V1−V2 regions were amplified comparably from 0.1 pg of the 16S rRNA_Mega plasmid and 3 pg of *E. coli* DH5α gDNA (Supplementary Fig. S6A). ORN_Mega suppressed DNA amplification from the 16S rRNA_Mega plasmid but not *E. coli* DH5α gDNA (Supplementary Fig. S6B). The 16S rRNA_Mega plasmid (0.1 pg) and *E. coli* DH5α gDNA (3 pg) were mixed and subjected to PCR in the presence or absence of ORN_Mega. Although the *16S rRNA* V1−V2 regions were amplified in both conditions (Supplementary Fig. S6B), DNA sequencing analysis of the PCR products showed that ORN_Mega suppressed DNA amplification only from the 16S rRNA_Mega plasmid (Supplementary Fig. S6C). We also confirmed that the DNA sequence corresponding to ORN_Mega was not present in the *16S rRNA* V1−V2 region in *E. coli* DH5α gDNA (Supplementary Fig. S6D). Thus, these results demonstrated the specificity of ORN_Mega.

Next, we performed ORNi-PCR with DNA extracted from human stool samples (Fig. 3C and D) and subjected the amplicons to NGS analysis. As shown in Fig. 3E, detection of the *Megamonas* sp. *rRNA* gene was reduced by one-third from 15.5 to 5.6%, demonstrating that ORNi-PCR suppressed the amplification of a *16S rRNA* gene in *16S rRNA* gene-based microbiome profiling, although suppression was incomplete. In addition, after suppression of amplification of the *16S rRNA* gene from *Megamonas* sp., detection of the *16S rRNA* gene from other major and some minor bacterial populations was increased (Fig. 3E and Supplementary Table S2). Similar results were obtained with DNA from another human stool sample using the same method (Supplementary Fig. S7). Thus, ORNi-PCR can suppress amplification of a targeted *16S rRNA* gene.

In gut microbiome profiling, host cell-derived mitochondrial *16S rRNA* is often a contaminant. In this regard, it was previously reported that blocking PCR with PNAs can suppress the amplification of host cell-derived *16S rRNA* (e.g. mitochondria or chloroplast *16S rRNA*) or *18S rRNA* sequences for higher resolution analysis of parasite microbes [11–13]. In addition, some bacteria-specific primers (e.g. 338R used in this study) can be used to amplify specifically bacterial *16S rRNA* genes without amplifying mitochondrial *16S rRNA* [11, 14]. If ORNi-PCR is used with such primers, amplification of the *16S rRNA* gene from the most abundant gut microbe population (i.e. *Bacteroides* sp.) can be suppressed, allowing other bacterial populations to be analyzed at higher resolution or detected *de novo* without increasing NGS read numbers (Supplementary Fig. S3). Such applications would be attractive, but further work (i.e. specific suppression of *Bacteroides 16S rRNA* genes) is needed to explore feasibility.

## Conclusions

In this study, we successfully applied ORNi-PCR to enrich target DNA sequences from a DNA mixture. We demonstrated the use of ORNi-PCR for analysis of different types of indel mutations introduced by genome editing, or bacterial populations in *16S rRNA* gene-based microbiome profiling. Thus, ORNi-PCR could prove useful in various fields including molecular biology and medical diagnosis.

## Author contributions

T.F. and H.F. conceived and designed the project. T.F. and D.M. performed experiments and analyzed data. T.F., D.M., and H.F. wrote the manuscript.

## Funding

This work was supported by Hirosaki University Graduate School of Medicine (T.F. and H.F.) and the Center of Innovation Program from the Japan Science and Technology Agency (H30W18; T.F. and D.M.).

## Conflict of interest statement

T.F. and H.F. have filed patent applications related to ORNi-PCR with the following details: (i) Method for suppressing amplification of specific nucleic acid sequences, Japanese Patent Application No. 2014-176018; (ii) Method for detecting differences in target nucleic acid region, Japanese Patent Application No. 2018-81752. T.F. and H.F. are founders and directors of Epigeneron Inc. and own shares in the company.

## Supplementary Material

bpz009_Supplementary_Data
