# Supplementary material for: Target enrichment from a DNA mixture by oligoribonucleotide interference-PCR (ORNi-PCR)
Source: Biol Methods Protoc. 2019 Aug 1;4(1):bpz009. doi: 10.1093/biomethods/bpz009 (PMC7200947; doi:10.1093/biomethods/bpz009)
Supplement: bpz009_Supplementary_Data [file bpz009_supplementary_data.zip › SupplFigure_20190711.pdf]

A

```
>hTHYN1   AAGTCCCCTGCAGCGTGACCATGTCGAGACCCCGGAAGAGGCTGGCTGGGACTTCTGG
>KO       AAGTCCCCTGCAGCGTGACCATGTCGAGACCCCGGAAGAGGCTGGCTGGGACTTCTGG
>KO       AAGTCCCCTGCAGCGTGACCATGTCGAGACCCCGGAAGAGGCTGGCTGGGACTTCTGG
>KO       AAGTCCCCTGCAGCGTGACCATGTCGAGACCCCGGAAGAGGCTGGCTGGGACTTCTGG
>KO       AAGTCCCCTGCAGCGTGACCATGTCGAGACCCCGGAAGAGGCTGGCTGGGACTTCTGG
>KO       AAGTCCCCTGCAGCGTGACCATGTCGAGACCCCGGAAGAGGCTGGCTGGGACTTCTGG
>KO       AAGTCCCCTGCAGCGTGACCATGTCGAGACCCCGGAAGAGGCTGGCTGGGACTTCTGG
>KO       AAGTCCCCTGCAGCGTGACCATGTCGAGACCCCGGAAGAGGCTGGCTGGGACTTCTGG
>KO       AAGTCCCCTGCAGCGTGACCATGTCGAGACCCCGGAAGAGGCTGGCTGGGACTTCTGG
>KO       AAGTCCCCTGCAGCGTGACCATGTCGAGACCCCGGAAGAGGCTGGCTGGGACTTCTGG
>KO       AAGTCCCCTGCAGCGTGACCATGT----ACCCCGGAAGAGGCTGGCTGGGACTTCTGG
>KO       AAGTCCCCTGCAGCGTGACCATGCCGAGACCCCGGAAGAGGCTGGCTGGGACTTCTGG
                                    AG
```

B

```
>hTHYN1   AAGTCCCCTGCAGCGTGACCATGTCGAGACCCCGGAAGAGGCTGGCTGGGACTTCTGG
>KO_ORN   AAGTCCCCTGCAGCGTGACCATG-C----CCCCGGAAGAGGCTGGCTGGGACTTCTGG
>KO_ORN   AAGTCCCCTGCAGCGTGACC-----------CCGGAAGAGGCTGGCTGGGACTTCTGG
>KO_ORN   AAGTCCCCTGCAGCGTGACC-----------CCGGAAGAGGCTGGCTGGGACTTCTGG
>KO_ORN   AAGTCCCCTGCAGCGAGACC-----------CCGGAAGAGGCTGGCTGGGACTTCTGG
>KO_ORN   AAGTCCCCTGCAGCGAGACC-----------CCGGAAGAGGCTGGCTGGGACTTCTGG
>KO_ORN   AAGTCCCCTG--------------------------------------GGACTTCTGG
>KO_ORN   ------------------------------CCCGGAAGAGGCTGGCTGGGACTTCTGG
```

Supplementary Figure S1

A

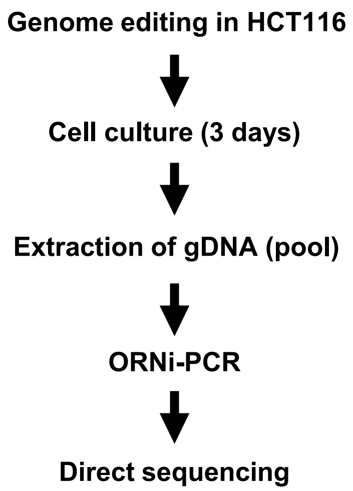

B

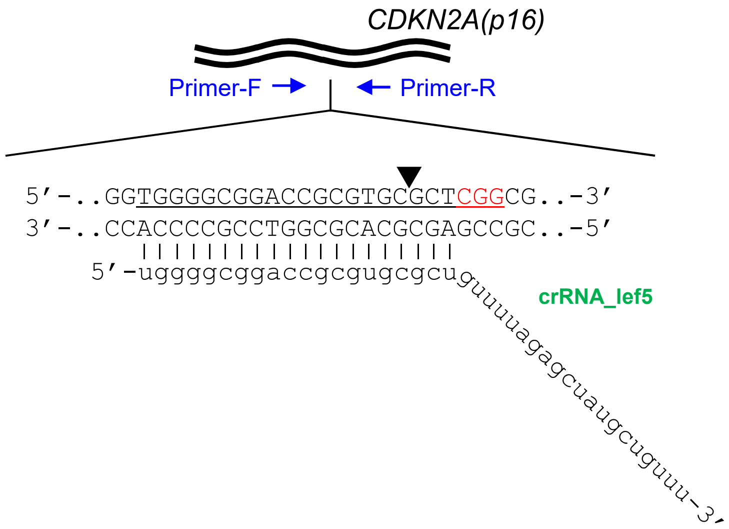

C

Temperature	Time	Cycle
94°C	2 min	1
98°C	10 s	34
74°C	1 min 10 s	

D

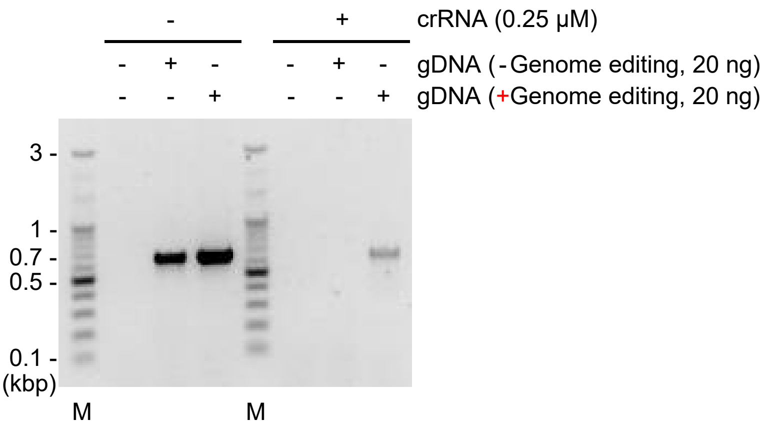

E

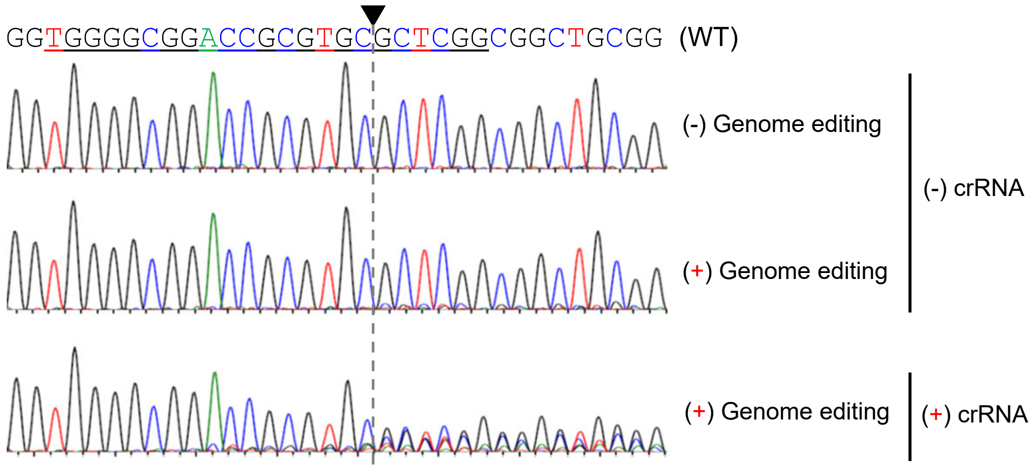

Supplementary Figure S2

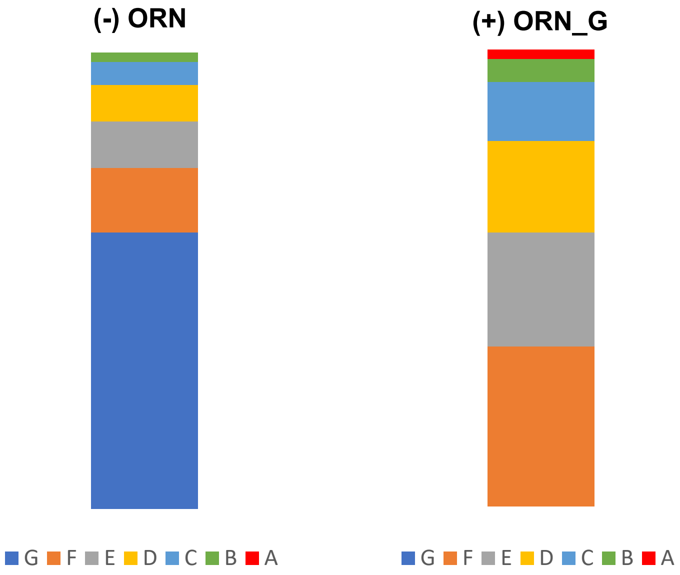

Supplementary Figure S3

A

Human stool

↓

DNA extraction

↓

PCR

↓

NGS

B

Temperature	Time	Cycle
94ºC	2 min	1
98ºC	10 s	30
55ºC	1 min	

C

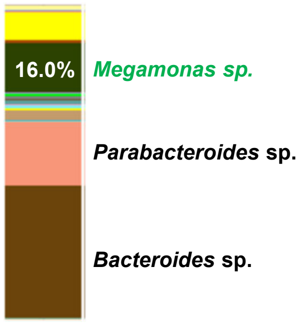

Supplementary Figure S4

A

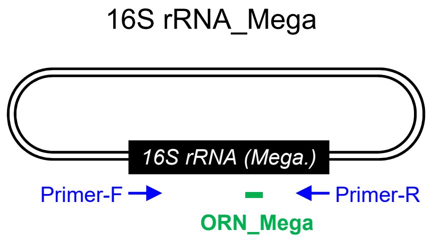

B

AGGGTTTGATTATGGCTCAGGACGAACGCTGGCGGCG
TGCTTAACACATGCAAGTCGAACGGGGTGTTTATTTC
GGTAAACACCAAGTGGCGAACGGGTGAGTAACGCGTA
AGCAATCTACCTTCAAGATGGGGACAACACTTCGAAA
GGGGTGCTAATACCGAATGAATGTAAGAGTATCGCAT
GAGACACTTACTAAAGGAGGCCTCTGAAAATGCTTCC
GCTTGAAGATGAGCTTGCGTCTGATTAGCTAGTTGGT
GAGGGTAAAGGCCCACCAAGGCGACGATCAGTAGCCG
GTCTGAGAGGATGAACGGCCACATTGGGACTGAGACA
CGGCCCAGACTCCTACGGGAGGCAGCA

C

Temperature	Time	Cycle
94ºC	2 min	1
98ºC	10 s	30
50, 55, or 60ºC	1 min	

D

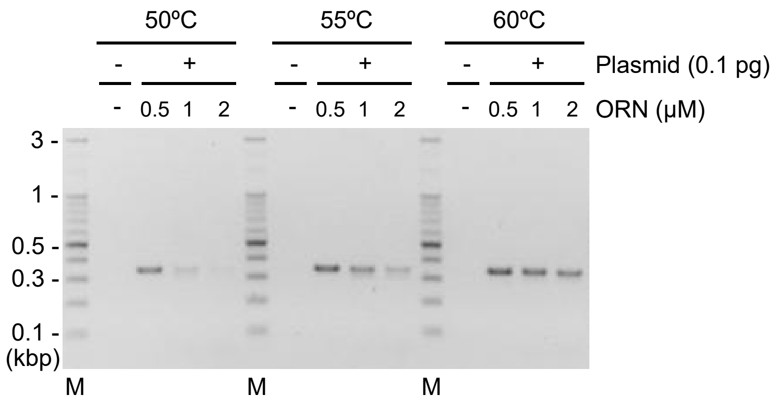

Supplementary Figure S5

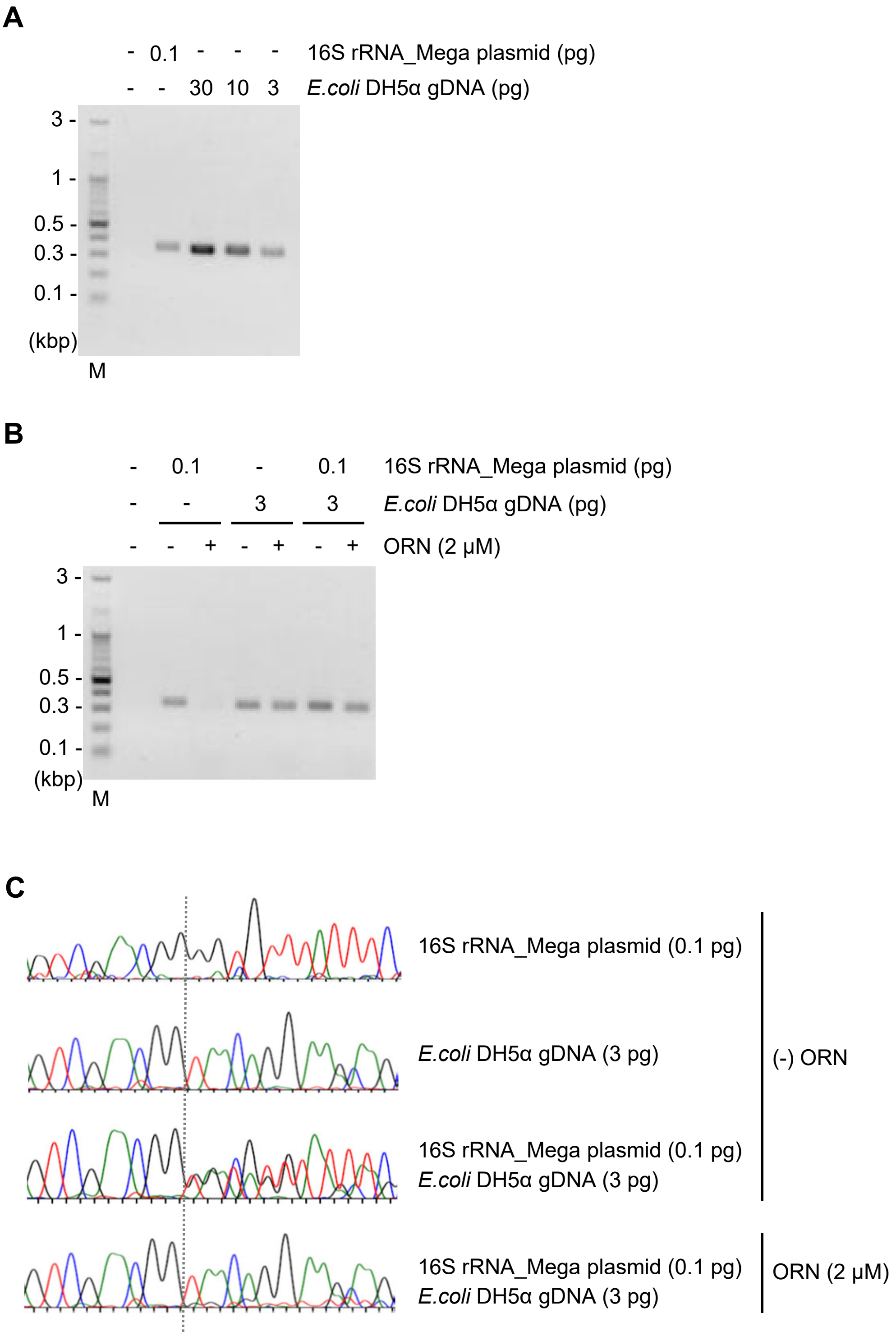

D

```
>Megamonas      CCTTCAAGATGGGGACAACACTTCGAAAGGGGTGCTAATACCGAATGAATGTAAGAGTAT
>DH5a           CCTGATGGAGGGGGATAACTACTGGAAACGGTAGCTAATACCGCATAA---------CGT
                ***    ** ***** ***   * **** **  ********** ** *           *

>Megamonas      CGCATGAGACACTTACTAAAGGAGGCCTCTGAAAATGCTTCCGCTTGAAGATGAGCTTGC
>DH5a           CGCAAGA-----CCAAAGAGGGGGACCTTCGGGCCTCTTGCCATC---GGATGTGCCCAG
                **** **       *   * ** * ***  *    *  * **       **** **    

>Megamonas      GTCTGATTAGCTAGTTGGTGAGGGTAAAGGCCCACCAAGGCGACGATCAGTAGCCGGTCT
>DH5a           ATGGGATTAGCTAGTAGGTG-GGGTAACGGCTCACCTAGGCGACGATCCCTAGCTGGTCT
                 *  *********** **** ****** *** **** ***********  **** *****
```

Supplementary Figure S6

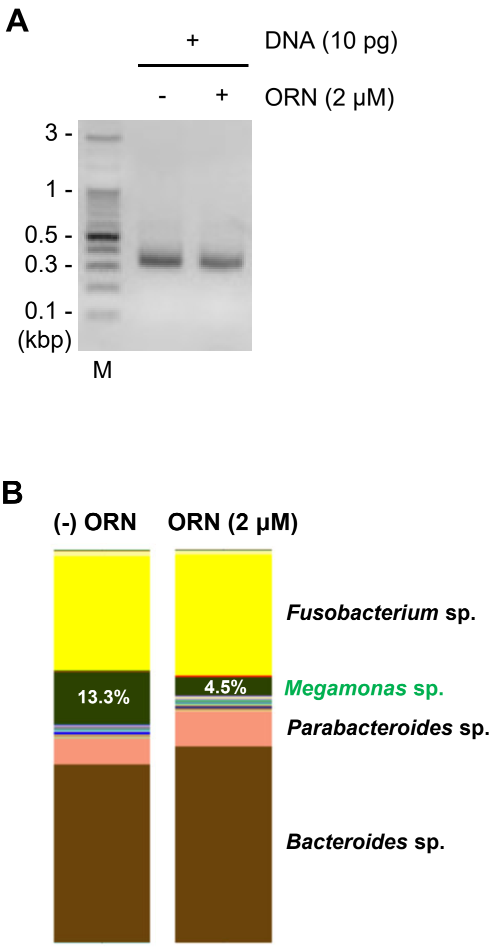

Supplementary Figure S7
