# Supplementary material for: Target enrichment from a DNA mixture by oligoribonucleotide interference-PCR (ORNi-PCR)
Source: Biol Methods Protoc. 2019 Aug 1;4(1):bpz009. doi: 10.1093/biomethods/bpz009 (PMC7200947; doi:10.1093/biomethods/bpz009)
Supplement: bpz009_Supplementary_Data [file bpz009_supplementary_data.zip › 190613 Supplementary_Text_rev.docx]

**Supplementary Text**

**Target enrichment from a DNA mixture by oligoribonucleotide interference-PCR (ORNi-PCR)**

Toshitsugu Fujita, Daisuke Motooka, Hodaka Fujii

**Supplementary Table and Figure Legends**

**Supplementary Table S1. Oligonucleotides used in this study**

**Supplementary Table S2. Number of bacterial *16S rRNA* reads detected in metagenome analysis**

**Supplementary Figure S1. Indel mutations introduced by genome editing.**

(**A**) Results of DNA sequencing for cloned PCR products. (**B**) Results of DNA sequencing for cloned ORNi-PCR products. PCR and ORNi-PCR products in Figure 2D (with genome editing) were cloned into plasmids, and their DNA sequences were analyzed by Sanger sequencing. CRISPR/Cas9 recognition sequences and cleavage sites are underlined and indicated by arrowheads, respectively. The PAM is shown in red.

**Supplementary Figure S2. Enrichment of genome-edited DNA sequences by ORNi-PCR with crRNA.**

(**A**) Schematic diagram of the analysis of genome-edited DNA sequences by ORNi-PCR. (**B**) Target position for genome editing and crRNA_lef5 targeting the human *CDKN2A (p16)* locus, used instead of an ORN. Forward and reverse sequences of gDNA are shown. The CRISPR/Cas9 recognition sequence and cleavage sites are underlined and indicated by arrowheads, respectively. The PAM is shown in red. (**C**) Conditions for Two-Step ORNi-PCR. (**D**) Results of ORNi-PCR. M, molecular weight markers. (**E**) DNA sequencing analysis of PCR and ORNi-PCR products. PCR or ORNi-PCR products in (**D**) were purified and subjected to Sanger sequencing using a forward primer.

**Supplementary Figure S3. Schematic diagram showing potential application of ORNi-PCR to *16S rRNA* gene-based microbiome profiling.**

Minor *16S rRNA* populations could be analyzed at higher resolution by suppressing the amplification of *16S rRNA* genes from more abundant *16S rRNA* populations (G) by ORNi-PCR. In addition, some *16S rRNA* populations could be detected *de novo*.

**Supplementary Figure S4. *16S rRNA* gene-based microbiome profiling.**

(**A**) Schematic diagram showing *16S rRNA* gene-based microbiome profiling. (**B**) Conditions for PCR. (**C**) Results of metagenome analysis. Detailed data are listed in Supplementary Table S2.

**Supplementary Figure S5. Evaluation of ORN_Mega.**

(**A**) Schematic diagram of ORNi-PCR with a 16S rRNA_Mega plasmid containing the V1−V2 region of the *Megamonas* sp. *16S rRNA* gene. (**B**) The DNA sequence of the V1−V2 region of *Megamonas* sp. *16S rRNA* gene was cloned into the 16S rRNA_Mega plasmid. The DNA sequence complementary to ORN_Mega is highlighted in green. Primer positions are shown in red. (**C**) Conditions for Two-Step ORNi-PCR. (**D**) Results of ORNi-PCR. M, molecular weight markers.

**Supplementary Figure S6. Specificity of ORN_Mega.**

(**A**) PCR with different amounts of *E. coli* DH5α gDNA. (**B**) Results of ORNi-PCR with ORN_Mega. (**C**) Results of DNA sequencing analysis. PCR and ORNi-PCR products purified from (**B**) were analyzed by Sanger sequencing. Regions of sequence signals are shown. The dotted line indicates the border between common and species-specific DNA sequences. (**D**) Comparison of DNA sequences of *16S rRNA* V1−V2 regions. DNA sequences surrounding the DNA sequence complementary to ORN_Mega (green circle) are shown. (**A** and **B**) PCR and ORNi-PCR were performed as shown in Figure 3C. M, molecular weight markers.

**Supplementary Figure S7. Suppression of target *16S rRNA* amplification by ORNi-PCR.**

(**A**) Results of ORNi-PCR. PCR and ORNi-PCR were performed as shown in Figure 3C with DNA from a different human stool sample. M, molecular weight markers. (**B**) Results of NGS analysis. PCR and ORNi-PCR products in (**A**) were purified and subjected to NGS analysis.
