# Supplementary material for: Target enrichment from a DNA mixture by oligoribonucleotide interference-PCR (ORNi-PCR)
Source: Biol Methods Protoc. 2019 Aug 1;4(1):bpz009. doi: 10.1093/biomethods/bpz009 (PMC7200947; doi:10.1093/biomethods/bpz009)
Supplement: bpz009_Supplementary_Data [file bpz009_supplementary_data.zip › 190617 SupplementaryProtocol.docx]

**Supplementary Protocol**

**Target enrichment from a DNA mixture by oligoribonucleotide interference-PCR (ORNi-PCR)**

Toshitsugu Fujita, Daisuke Motooka, Hodaka Fujii

**Materials and Reagents**

1. 1.5 ml centrifuge tube
2. 0.2 ml PCR tubes
3. KOD -Plus- Ver.2 (KOD-211, Toyobo)
4. Oligoribonucleotides (FASMAC): Dilute to a concentration of 10 µM with DNase/RNase-free water.

**Equipment**

1. Pipettes
2. Thermal cycler

**Procedure**

ORNi-PCR is performed as described previously [4]. An example is shown below.

1. Design of ORNs

Length: 17–25 bases

Position: Positions between a primer set can be flexibly chosen.

Prediction of Tm: (a + u) * 2 + (g + c) * 4, in which a, u, g, and c are the numbers of the bases A, U, G, and C, respectively.

1. ORNi-PCR Reactions
   1. Prepare 10 µl of reaction mixture as follows:

|  |  | with ORN | without ORN |
| --- | --- | --- | --- |
| Reagents |  | µl | µl |
| Template ^a^ |  | 1.0 | 1.0 |
| 10× Buffer for KOD -Plus- Ver.2 |  | 1.0 | 1.0 |
| 2 mM dNTPs |  | 1.0 | 1.0 |
| 25 mM MgSO_4_ |  | 0.6 | 0.6 |
| Primer-F (10 µM) |  | 0.3 | 0.3 |
| Primer-R (10 µM) |  | 0.3 | 0.3 |
| ORN (10 µM) |  | 0.5–2.0 ^b^ | - |
| KOD -Plus- Ver.2 (1 U/µl) |  | 0.2 | 0.2 |
| DDW |  | to 10.0 | to 10.0 |

^a^: Mammalian genomic DNA (20 ng/µl), *E. coli* genomic DNA (3 pg/µl), plasmid DNA (0.1 pg/µl), and human stool DNA (10 pg/µl) were used as templates in this study.

^b^: The optimal concentration is between 0.5–2.0 µM [4]. As shown in Supplementary Figure S5, the optimal ORN concentration is found by testing several ORN concentrations. Excess amounts of ORNs may result in non-specific inhibition of PCR reactions.

- 1. Perform two-step ORNi-PCR reactions as follows:

| Temperature | Time | Cycle |
| --- | --- | --- |
| T = 94°C | 2 min | 1 |
| T = 98°C | 10 sec | 30–35 |
| T = X°C ^c^ | 1 min / kbp + 30 sec |  |

^c^: Annealing and elongation are performed together in a single step. As shown in Supplementary Figure S5, the optimal temperature is found by testing several temperatures around the predicted Tm of an ORN.

- 1. Evaluate amplifications using standard agarose gel electrophoresis.
